# Incidence of Distal Stent Graft Induced New Entry vs. Aortic Remodeling Associated With Frozen Elephant Trunk

**DOI:** 10.3389/fcvm.2022.875078

**Published:** 2022-03-10

**Authors:** Matti Jubouri, Fatima Kayali, Priyanshu Saha, Daniyal M. Ansari, Yousef Rezaei, Sven Z. C. P. Tan, Mostafa Mousavizadeh, Saeid Hosseini, Idhrees Mohammed, Mohamad Bashir

**Affiliations:** ^1^Hull York Medical School, University of York, York, United Kingdom; ^2^School of Medicine, University of Central Lancashire, Preston, United Kingdom; ^3^School of Medicine, St George's University of London, London, United Kingdom; ^4^Heart Valve Disease Research Center, Rajaie Cardiovascular Medical and Research Center, Iran University of Medical Sciences, Tehran, Iran; ^5^Barts and The London School of Medicine and Dentistry, Queen Mary University, London, United Kingdom; ^6^Institute of Cardiac and Aortic Disorders (ICAD), SRM Institutes for Medical Science (SIMS Hospital), Chennai, India; ^7^Vascular and Endovascular Surgery, Velindre University NHS Trust, Health Education and Improvement Wales (HEIW), Cardiff, United Kingdom

**Keywords:** aortic dissection, aortic aneurysm, aortic surgery, frozen elephant trunk, total arch replacement

## Abstract

**Background:**

The introduction of the frozen elephant trunk (FET) technique for total arch replacement (TAR) has revolutionized the field of aortivascular surgery by allowing hybrid repair of complex aortic pathologies in a single step through combining an open surgical approach with an endovascular one. FET has been associated with favorable aortic remodeling, however, its is also associated with development of distal stent graft induced new entry (dSINE) tears postoperatively. The rate of aortic remodeling and the incidence of dSINE have been linked together, in addition, there seems to be a relationship between these two variables and FET insetion length as well as graft size.

**Aims:**

The scope of this review is to highlight the rate of aortic remodeling as well the incidence of dSINE associated with different FET devices available commercially. This review also aimed to investigate the relationship between aortic remodeling, dSINE, FET insertion length and FET graft size.

**Methods:**

We conducted a comprehensive literature search using multiple electronic databases including PubMed, Ovid, Scopus and Embase in order to collate all research evidence on the above mentioned variables.

**Results:**

Thoraflex™ Hybrid Plexus seems to yield optimum aortic remodeling by promoting maximum false thrombosis as well true lumen expansion. Thoraflex Hybrid™ is also associated with the lowest incidence of dSINE post-FET relative to the other FET devices on the market. Aortic remodeling and dSINE do influence each other and are both linked with FET graft length and size.

**Conclusion:**

The FET technique for TAR shows excellent aortic remodeling but is associated with a considerable risk of dSINE development. However, Thoraflex™ Hybrid has demonstrated itself to be the superior FET device on the aortic arch prostheses market. Since aortic remodeling, dSINE, FET insertion length and stent graft size are all interconnect, the choice of FET device length and size must be made with great care for optimum results.

## Introduction

The frozen elephant trunk (FET) technique for total arch replacement (TAR) was introduced in 2003 and has been in use to treat a wide range of complex aortic pathologies (1). This marked an evolution from the two-stage elephant trunk (ET) procedure which involved replacing the ascending aorta and arch with a graft, followed by placement of an “elephant trunk” prosthetic that extended into the descending aorta. The conventional ET impeded full aortic remodeling and potentially caused injury to the aortic intima, thus negatively influencing clinical outcomes. Therefore, necessitating a second surgical intervention to either extend the graft or attach it to the relative aortic segment (2). Hence, FET ameliorated the two-stage procedure through innovation and permitted combined open surgical replacement of the aortic arch along with endovascular intervention in the descending aorta during a single operation in hybrid theaters.

The FET procedure has been associated with favorable aortic remodeling outcomes (3, 4). Aortic remodeling, however, has had a multitude of definitions across the literature. Different studies have calculated this significant prognostic tool in varying ways; however, it is essentially a late effect characterized by the occlusion of the primary entry tear site subsequently promoting false lumen thrombosis. This led to a decrease in false lumen (FL) diameter and a re-expansion of the true lumen (TL) (5). It is well-established within the literature that aortic remodeling has proved to be an accurate prognostic tool for patients (6). Additionally, aortic remodeling has been linked to improved survival rates and form an overall good measure of clinical success (7). Failure of false lumen thrombosis has been linked to aneurysm dilatation, rupture of the thoracic aorta, and higher reintervention rates (8).

One of the noted complications of FET is development of distal stent graft induced new entry (dSINE) tears, typically due to the use of endovascular manipulation as well as stent graft and distal TL size mismatch. The choice of FET devices as well as its size and length has shown to be linked with the incidence of dSINE. Evidence in the literature also suggest that there is an inverse relationship between dSINE and the rate of aortic remodeling distally (9).

This review aimed to highlight the incidence of distal stent graft induced new entry (dSINE) as well as the rate of aortic remodeling associated with TAR with FET. Another scope of this review was to assess the efficacy of the Thoraflex™ Hybrid device in achieving optimal remodeling and dSINE outcomes. Finally, we investigated the relationship between aortic remodeling, dSINE, and FET graft length and size.

## Aortic Remodeling

The revolutionary FET procedure is well-established in the literature with excellent aortic remodeling, including favorable FL thrombosis rates and significant positive changes in TL and FL diameters (3, 4). A 2019 study on total arch replacement reported that using the FET technique proved to have a high rate of TL expansion and FL thrombosis at the level of bronchial carina, in addition to the descending aorta. It was also thought that FET reduced the sequalae of vascular complications resulting from failed aortic remodeling (3). These results are also not expected to differ between acute and chronic aortic dissection presentations (10). In a meta-analyis that included 1,279 patients, FL thrombosis was achieved in 96.8% of patients (95% CI, 90.7–98.9%) (11). Similarly, these findings were supported in a review by Di Bartolomeo et al. (12), where complete or partial FL thrombosis was expected in 90% of patients. This figure is incomparable to conservative management, wherein FL thrombosis occurred in between 33.3 and 77.8% of patients (12). Unfortunately, these promising results have not been seen to the same extent at the level of the abdominal aorta and further management has been recommended for this subset (10).

The Thoraflex™ Hybrid Plexus (THP™) (Terumo Aortic, Inchinnan, Scotland, UK) device has been designed with a special interrupted pattern, which is thought to protect the aortic wall from the substantial forces of blood flow to achieve excellent aortic remodeling (13). Mehanna et al. (13), who investigated aortic remodeling following FET with THP™, carried out Spearman rank correlation tests to assess the correlation of their results. Aortic remodeling ratios, prior to and following the procedure, had a moderately positive correlation. This was calculated as 0.566 and 0.582 for TL expansion and FL diameter decrease, respectively, with *p* values of 0.006 and 0.004, respectively, indicating strong statistical significance. A significant expansion of the TL ratio was seen using Thoraflex™ Hybrid post-operatively, with a median increase from 0.31 to 0.4 mm (*p* = 0.042). This trend matched the significant FL ratio decrease from 0.66 to 0.54 mm (*p* = 0.02). The study included a total on 20 patients out of whom 8 were acute and 12 were chronic, with no significant difference found when comparing aortic remodeling in both groups (*p* = 0.26). The authors stressed that Thoraflex™ Hybrid is the optimal FET device as it is the safest to use due to its interrupted pattern as mentioned earlier (13).

In the previous studies described, the authors used a Computed Tomography (CT) scan to assess the aortic remodeling diameters. In a 2021 study by Usai et al. (14), volume measurements were used instead to overcome the 2D view limitations of CT images, allowing the authors to gauge a more accurate measure of lumen patency using the Thoraflex™ Hybrid device. Here, surfaces and volumes were measured using CT angiography prior to and following FET, in addition to 12- and 24-month follow-up. The mean true lumen (in cm^3^) prior to the FET was 77.03 cm^3^ (± 47.96 cm^3^). At discharge, this mean increased by 10.96 cm^3^. This growth was sustained and the TL measurements 12 and 24 months after were 113.83 and 133.84 cm^3^, respectively. This long-term analysis of TL volumetric expansion evidenced a statistically significant growth even after 2 years of the FET procedure (*p* = 0.047). Similarly, the mean FL measurement was 158.33 cm^3^ (± 68.24 cm^3^), 167.56 cm^3^ (± 90.24 cm^3^), 164.36 cm^3^ (± 59.72 cm^3^) and 157.20 cm^3^ (± 78.0 cm^3^) prior to the procedure, following and at 12-month and 24-month follow-up (14). These long-term outcomes seem to be congruent a previous 2018 study, where chronic dissection patients showed significant TL expansion (15 ± 17 mm to 28 ± 2 mm) (*p* = 0.001) and FL diameter drop (40 ± 11 mm to 32 ± 17 mm) (*p* = 0.026) 2 years following the procedure (15). Additionally, Usai et al. (14) confirmed that in their study almost all Thoraflex Hybrid™ patients had significant TL expansion and reported only 1 patient required reintervention, due to a FL aneurysm. Furthermore, the authors also noted that the most significant growth in the surface TL measurement was at the level of the diaphragm (*p* = 0.00193) (14).

There appears to be some controversy in the literature regarding the impact of presentation on the aortic remodeling following FET procedures. It was initially expected that there is no difference between acute and chronic dissections, however, in a study involving 100 patients who had Thoraflex Hybrid™ implanted, FL thrombosis was achieved at a much more accelerated pace in acute, rather than chronic dissection patients. Despite this, full FL thrombosis was achieved in 100% of patients within 24 months at the proximal segment of the aorta. Additionally, as expected, distal segments of the aorta had less successful rates (16). An international multicentre registry which used E-Vita in 137 patients (65 acute and 72 chronic) also confirmed that acute dissections are associated with greater aortic remodeling rates (10). When comparing the FL thrombosis rates in both studies together, Thoraflex™ Hybrid came out on top with improved results.

As reported by Shrestha et al. (17), the 100% rate of FL thrombosis achieved using Thoraflex™ shows its superiority to other devices as seen by the 82.1% and 89.4% rates reported by Kobayashi et al. (16) and Chen et al. (18), respectively. Yet it is important to take into consideration the differences amongst these 3 independent studies. Kobayashi et al. (16) carried out a simplified FET technique in 34 patients with complicated acute type A aortic dissections. The “simplified” approach involves using gelatine-resorcinol adhesive to attach the dissected aortic arch wall layers, followed by an antegrade open arch approach to deploy the aortic stent-graft into the arch or proximal descending aorta. Post-operatively, only 82.1% of patients had FL thrombosis seen in the aortic arch using the TAG, Talent and E-Vita stent-grafts (16). On the other hand, Chen et al. (18) reported complete FL thrombosis in 89.4% of patients around the triple-branched stent-graft, created by Yuhengjia Science and Technology. In this study, 122 patients underwent total arch replacement for acute type A aortic dissection, with open placement of this triple-branched stent graft (18). Similarly, in a study involving 41 acute type A dissection patients who underwent TAR with FET using E-Vita, a FL thrombosis rate of 93.9% at the pulmonary trunk level was achieved (19). The above evidence shows beyond doubt that Thoraflex Hybrid™ offers the highest rate of FL thrombosis.

The Thoraflex™ Hybrid Plexus has also demonstrated TL expansion not only at the level of pulmonary bifurcation (covered by the stent), but also at the distal descending aorta, with complete FL thrombosis in 73.1% of cases at the former site (20). Similar to other findings in the literature, there was a small, albeit significant, expansion of aortic diameter at the level of the abdominal aorta. Further benefit yielded Terumo Aortic's Thoraflex™ Hybrid was highlighted in a study by Fiorentino et al. (20), where pre-planned second-stage procedures were prevented in 18.2% of patients due to the promising aortic remodeling rates. Additionally, when comparing Thoraflex™ Hybrid to other devices, the need for endoprosthetic extensions due to incomplete FL thrombosis was less by 6% with the Thoraflex™ Hybrid in comparison with the E-Vita HP (21).

In a more detailed study on aortic remodeling after FET with Thoraflex Hybrid™ for acute (*n* = 31) and chronic dissections (*n* = 34) at the stent, thoracoabdominal transition and coeliac trunk levels, significant TL and FL changes were noted (15). A summary of the findings showing the favorable aortic remodeling associated with Thoraflex Hybrid™ is illustrated in Tables 1, 2.

**Table 1:** Summary of TL diameter results from Berger et al. (Thoraflex Hybrid) (15).

**Aortic Level**	**Dissection**	**FL Diameter (mm)**				
		**Preoperatively**	**6 months**	**12 months**	**24 months**	**36 months**
L1	Acute	23 ± 11	16 ± 10 (*p = * 0.14)	14 ± 8 (*p = * 0.16)	8 ± 3 (*p = * 0.019)	4 ± 2 (*p = * 0.53)
	Chronic	40 ± 11	29 ± 13 (*p < * 0.001)	32 ± 17 (*p = * 0.004)	32 ± 16 (*p = * 0.026)	24 ± 13 (*p = * 0.003)
L2	Acute	20 ± 11	20 ± 11 (*p = * 0.61)	18 ± 12 (*p = * 0.97)	13 ± 7 (*p = * 0.22)	12 ± 12 (*p = * 0.50)
	Chronic	29 ± 11	26 ± 12 (*p = * 0.03)	28 ± 13 (*p = * 0.062)	24 ± 7 (*p = * 0.001)	14 ± 11 (*p = * 0.059)
L3	Acute	15 ± 12	11 ± 11 (*p = * 0.35)	11 ± 9 (*p = * 0.69)	12 ± 10 (*p = * 0.11)	11 ± 11 (*p = * 0.50)
	Chronic	21 ± 9	22 ± 9 (*p = * 0.89)	22 ± 9 (*p = * 0.53)	27 ± 3 (*p = * 0.79)	15 ± 11 (*p = * 0.24)

*L1, segment at the stent level; L2, segment at the thoracoabdominal transition; L3, segment at the coeliac trunk*.

**Table 2:** Summary of FL diameter results from Berger et al. (Thoraflex Hybrid) (15).

**Aortic Level**	**Dissection**	**TL Diameter (mm)**				
		**Preoperatively**	**6 months**	**12 months**	**24 months**	**36 months**
L1	Acute	23 ± 12	28 ± 6 (*p = * 0.88)	28 ± 4 (*p = * 0.83)	27 ± 2 (*p = * 0.058)	27 ± 0 (*p = * 0.56)
	Chronic	15 ± 7	24 ± 6 (*p < * 0.001)	25 ± 5 (*p = * 0.002)	28 ± 2 (*p = * 0.001)	29 ± 2 (*p = * 0.063)
L2	Acute	17 ± 13	26 ± 11 (*p = * 0.22)	25 ± 7 (*p = * 0.60)	24 ± 10 (*p = * 0.097)	22 ± 8 (*p = * 0.21)
	Chronic	15 ± 8	18 ± 9 (*p = * 0.003)	22 ± 8 (*p = * 0.001)	27 ± 3 (*p = * 0.001)	28 ± 2 (*p = * 0.008)
L3	Acute	17 ± 11	21 ± 10 (*p = * 0.023)	20 ± 8 (*p = * 0.75)	18 ± 9 (*p = * 0.049)	17 ± 12 (*p = * 0.80)
	Chronic	14 ± 8	15 ± 5 (*p = * 0.035)	17 ± 7 (*p = * 0.069)	15 ± 6 (*p = * 0.27)	18 ± 8 (*p = * 0.47)

*L1, segment at the stent level; L2, segment at the thoracoabdominal transition; L3, segment at the coeliac trunk*.

The same aforementioned international multicentre registry which used E-Vita in 137 patients (65 acute and 72 chronic) also measured TL diameter preoperatively and at follow-up, which had a median of 32 months (21–53 months) (10). A summary of the results can be found in Table 3. Additionally, in a different study using the Cronus device, authors reported no change in TL diameter at the level of the aortic arch (22). Comparing the results from the three devices, Thoraflex Hybrid™ clearly yields the optimum aortic remodeling.

**Table 3:** Summary of TL diameter results from Iafrancesco et al. (E-Vita) (10).

**Aortic Level**	**Dissection**	**TL Diameter (mm)**		
		**Preoperatively**	**Follow-up**	***p*-value**
L1	Acute	26 (20–30)	28 (25–30)	0.009
	Chronic	29 (25–33)	30 (26–32)	0.448
L2	Acute	25 (20–30)	27 (24–30)	<0.001
	Chronic	25 (21–30)	27 (25–30)	0.082
L3	Acute	24 (19–28)	26 (22–29)	0.002
	Chronic	25 (21–28)	26 (24–29)	<0.001
L4	Acute	22 (17–26)	24 (19–27)	0.014
	Chronic	26 (22–29)	25 (22–29)	0.261

*L1, stent graft level; L2, just below the end of the stent graft; L3, 10th thoracic vertebra level; L4, coeliac artery level*.

Variations of reported aortic remodeling data in the literature could be attributed to multiple factors. For instance, differences in results could be a result of the varying pathologies that the FET procedure is used to treat, in addition to the heterogenous population included in these studies. Such variance in demographics and comorbidities, such as advanced age, can have a negative prognostic effect on the procedure outcomes (14). It is also important to note that there was an evident lack of large cohort, multicentre trials and comparative studies involving the Thoraflex Hybrid™ device and further studies must take place to evidence the promising results of this novice tool. Table 4 Summarizes all the findings in Distal Stent Induced New Entry.

**Table 4:** Summary of studies included in Distal Stent Induced New Entry and their aortic remodeling data reported.

**References**	**Year**	**Cohort**	**Acute/Chronic**	**HP(s) Used**	**Stent Size**	**Outcomes**			
						**TL Diameter**	**FL Diameter**	**FL thrombosis**	**Other findings**
Mehanna et al. (13)	2021	22	8 acute 14 chronic	Thoraflex	Median Stent outer diameter was 28 mm (24–40 mm) and length was 125 mm (100–150 mm).	Significant increase in the TL: total aortic diameter ratio (*P* = 0.031), with a median increase from 0.31 to 0.4 mm.	Significant decrease in the FL: total aortic diameter ratio (*P* = 0.024), with a median drop from 0.66 to 0.54 mm.	N/A	No significant changes in aortic remodeling between acute and chronic (*p = * 0.26).
Usai et al. (14)	2021	20	Not specified	Thoraflex	Not specified	Mean TL (in cm^3^): **Pre-operatively**: 77.03, SD 47.96 **At discharge**: 87.99, SD 33.98 **12-months**: 113.83, SD 37.33 **24-months**: 133.84, SD 28.10 (*p = * 0.04)	Mean FL (in cm^3^): **Pre-operatively:** 158.33, SD 68.24 **At discharge:** 167.56, SD 90.24 **12-months:** 164.36, SD 59.72 **24-months:** 157.20, SD 78.0	N/A	The most significant growth in the surface TL measurement was at the level of the diaphragm (*p = * 0.00193).
Berger et al. (15)	2018	65	Acute (A): 31 Chronic (C): 34	Thoraflex	**Length:** 100 mm	**At the stent level:** C: from 15 ± 7 mm to 28 ± 2 mm after 24 months (*p = * 0.001). A: from 23 ± 11 mm to 8 ± 3 mm (*p* = 0.019) **At thoracoabdominal transition**: C: from 15 ± 8 mm to 28 ± 2 mm after 36 months (*p* = 0.008) A: from 17 ± 13 mm to 23 ± 8 mm (*p* = 0.01) postoperatively **At the coeliac trunk** (after 6 months): C: from 14 ± 8 mm to 15 ± 5 mm (*p* = 0.035) A: 17 ± 11 mm to 21 ± 10 mm (*p* = 0.023)	**At the stent level:** C: from 40 ± 11 mm to 24 ± 13 mm after 36 months (*p* = 0.003) **At thoracoabdominal transition**: C: 29 ± 11 mm to 24 ± 7 mm after 24 months (*p* = 0.001) **At the coeliac trunk**: A: 15 ± 12 mm to 11 ± 9 mm (*p* = 0.001) postoperatively.	N/A	N/A
Fiorentino et al. (20)	2021	28	Chronic: 28	Thoraflex	The **proximal graft diameter**: between 26 and 30 mm. The **distal stent graft diameter** between 28 and 40 mm. **Length**: 150 mm in 19 cases (67.9%), and 100 mm in 9 patients (32.1%).	Mean **proximal descending aorta**: increased from 13.0 (±6.4) to 23.4 (±7.8) (*p < * 0.001) **Distal descending aorta**: from 12.1 (±4.7) to 19.1 (±9.8) (*p = * 0.009) **Abdominal aorta**: from 10.8 (±2.9) to 11.5 (±3.1).	Mean **proximal descending aorta**: decreased from 37.2 (±12.2) to 27.1 (±16.7) (*p = * 0.012) **Distal descending aorta**: from 32.7 (±13.9) to 25.5 (±13.0) (*p = * 0.042) **Abdominal aorta**: from 17.2 (±5.9) to 17.8 (±5.6).	**Proximal descending aorta**: partial in 3 patients (11.5%) and complete thrombosis in 19 (73.1%). **Distal descending aorta:** 3 partial (12.5%) and complete thrombosis in 10 (41.7%). **Abdominal aorta**: partial in 1 (4.2%).	N/A
						**TL Diameter**	**FL Diameter**	**FL thrombosis**	**Other findings**
Di Marco et al. (21)	2020	318	Acute: 119 Chronic: 25 Other indications: 174	E-Vita (173) Thoraflex (145)	**Not specified**	N/A			Endoprosthetic extensions were performed (often for incomplete FL thrombosis) in: E-Vita: 45 patients Thoraflex: 40 patients
Shrestha et al. (17)	2016	100	Acute: 37 Chronic: 31 Aneurysms: 32	Thoraflex	**Diameter** of stented portion: 26- 40 mm. The **length** of the stented part was either 100 or 150 mm.	N/A		100% achieved within 24 months.	FL thrombosis occurred much more quickly in acute, in contrast to chronic dissections.
Kobayashi et al. (16)	2016	34	Acute: 34	TAG: 6 Talent: 5 E-Vita: 23	**Mean diameters**: TAG: 34.5 ± 3.5 mm Talent: 34.2 ± 1.6 mm E-Vita: 32.4 ± 2.9 mm	Overall diameter of the aortic arch significantly reduced (*p < * 0.05).	Distal to the aortic stent, 30% (7/34) patients showed significant FL diameter decrease.	82.1% (23/34) at the aortic arch.	N/A
Chen et al. (18)	2013	122	Acute: 122	Triple-branched stent graft (Yuhengjia Science and Technology, Corp, Ltd, Beijing, China)	**Length**: 145 mm **Proximal diameter**: 30 or 32 mm **Distal diameter**: 26 or 28 mm	N/A		89.4% (101/113) at the stent level, 3-months follow-up.	N/A
Akbulut et al. (19)	2019	41	Acute: 41	E-Vita	**Not specified**	Aortic diameters increased from pre-operatively (37.4 ± 8.9) to postoperative (31.8 ± 4.9, *p < * 0.001) aortic diameters. The mean difference between the two groups was 5.60 mm (95% confidence interval [8.10–3.09])	N/A	93.9% at the pulmonary trunk level.	N/A
Iafrancesco et al. (10)	2017	137	Acute: 65 Chronic: 72	E-Vita	**Median [interquartile range] diameter:** 28 mm [24–30 mm].	Please refer to Table 3			
Song et al. (22)	2020	16	Acute: 16	Cronus	**Length:** 100–150 mm	Please refer to Figures in original article			
Panfilov et al. (23)	2021	44	Not specified	E-Vita	**Diameter:** 24–30 mm **Length:** 150 mm	N/A		Zone 2: 60% Zone 3: 77% (*p* = 0.046).	Reintervention rate: Zone 2:25.9% Zone 3: 8.3%
Berger et al. (24)	2019	88	Acute: 88	Thoraflex E-Vita	FET length: Thoraflex: 100 mm E-Vita: 130 mm	N/A		Thoraflex: 74% at 1-year follow-up	N/A

## Distal Stent Induced New Entry

As mentioned earlier, dSINE is a serious complication associated with FET where a new intimal entry tear develops distally due to the stent graft portion of the FET HP. Untreated dSINE has showed a mortality rate of up to 25% following endovascular aortic intervention (9, 25). The distal landing zone of the stent is often located in the diseased zone of the aorta, as a result, the stent graft portion of the FET device can induce injury to the intima of the aorta due to the fragile and mobile dissection membranes mismatching with a stiff stent graft (26, 27). dSINE necessitates secondary intervention which negates the benefit of FET being a 1-stage process. Nevertheless, secondary endovascular reintervention has shown to have excellent clinical outcome (25). Therefore, oversizing the FET graft is strongly suggested to increase the risk of dSINE forming, however, this has been challenged (25, 27, 28). This relationship between dSINE occurrence and the size of the graft will be discussed further on in this review. As will also be discussed later in this review, formation of dSINE perfuses the FL and can lead to a new patent FL, which significantly impede positive aortic remodeling (26, 29, 30).

The incidence of dSINE for any device is varied between different studies using the same device, this is likely to be associated with the difference in the study design, population (baseline morbidity), and surgical technique or device size and length used. For example, the incidence of dSINE reported in the studies identified ranged from 0 to 14.5% with Thoraflex Hybrid™, 1–18.2% with E-Vita, and 0–27.3% with Frozenix™. The size and length of device used are interconnected with the incidence of dSINE (9, 20, 24, 25, 31–37). A summary of all the dSINE findings to-be discussed in this section can be found in Table 5.

**Table 5:** Summary of results in Is Aortic Remodeling Correlated With dSINE?

**Study**	**Year**	**FET Cohort**	**FET Device**	**dSINE**
Kreibich et al. (9)	2020	126	Thoraflex™	13%
Charchyan et al. (32)	2021	20	Thoraflex™ (*n = * 3)	13%
			E-Vita™ (*n = * 13)	4.5%
			Valiant™ (*n = * 4)	23.5%
Tsagakis et al. (33)	2018	286	E-Vita™	1%
Berger et al. (24)	2019	88	Thoraflex™ (*n = * 55)	14.5%
			E-Vita™ (*n = * 33)	18.2%
Fiorentino et al. (20)	2020	28	Thoraflex™	7.10%
Hirano et al. (34)	2019	76	Frozenix™	4.00%
Yoshitake et al. (35)	2020	139	Custom-made Frozenix™	0.72%
Iino et al. (36)	2020	50	Frozenix™ and Gelweave™ combination	0%
Yamane et al. (37)	2020	15	Frozenix™	27.30%
Nomura et al. (25)	2021	70	Frozenix™	12.90%
Furutachi et al. (31)	2019	19	Frozenix™	15.80%

Terumo Aortic's Thoraflex™ Hybrid is an excellent FET device with very favorable survival outcomes that are to some extent superior to those offered by other commercially available devices. The incidence of dSINE with Thoraflex Hybrid™ can be considered the lowest within the aortic arch prostheses market as demonstrated above (9, 20, 24, 25, 31–37).

One of the many studies that reported a relatively low incidence of dSINE is Kreibich et al. (26), with 12.7% of patients developing dSINEs 27 months following FET with Thoraflex Hybrid™. Other than the aforementioned fact that the dSINE group of patients had smaller true lumen diameters at the level of the stent graft (L1; *p* = 0.251) and the level of thoracoabdominal transition (L2; *p* = 0.44), there was no difference in type of dissection or clinical characteristics between patients that did and did not develop dSINE. Another study by the same authors (26) showed the safety, efficacy and consistency of THP™ by reporting a dSINE rate of 11.4%.

Charchyan et al. (32) compared the rate of dSINE in different operative stent devices. Group one was treated with distal Z shaped nitinol stent grafts (E-Vita), group two was treated with ring shaped nitinol stent graft (Thoraflex™ Hybrid) and group three used distal dissection-specific stent grafts (Valiant™ retrograde stent graft, Medtronic Vascular, Santa Rosa, CA, USA). Upon univariate and multivariate risk factor analysis, the study determined two statistically significant predictors of developing dSINE, which included connective tissue disorder and stent graft diameter. Univariate risk factor analysis of connective tissue disorder being associated with developing dSINE was derived to have a hazard ratio of 4.02 (95% CI, 0.89–18.27; *p* = 0.072). Moreover, multivariate analysis showed connective tissue disorder to have a hazard ratio of 5.62 (95% CI, 0.68–46.59; *p* = 0.110). Univariate risk factor analysis of the association of stent graft diameter with developing dSINE showed to have a hazard ratio of 1.36 (95%CI,1.08–1.71; *p* = 0.009). Furthermore, multivariate analysis derived a hazard ratio of 1.37 (95% CI, 1.02–1.83; *p* = 0.034). This study is helpful as it provides significant evidence supporting the numerical trends suggested by Kreibich et al. (9). Charchyan et al. (32) also suggested using a dissection specific stent graft with a tapered structure thus preventing oversizing at the distal edge or using a longer stent graft to minise the occurrence of dSINE.

This study proved that with the E-Vita Z-shaped nitinol stent grafts, dSINE occurrence was significantly higher than the Thoraflex Hybrid™ ring shaped nitinol stent graft. In the E-Vita group, 13% of patients developed dSINE whereas in the Thoraflex Hybrid™ group this was only 4.5%, which was significantly less (*p* = 0.043, Cramer's V = 0.24). The mean follow up time was 16 months and 14.5 months respectively for group one and two. This study contradicts Kreibich et al. (9) study, as it provides a direct comparison between devices and confirms Thoraflex Hybrid™ superiority over E-Vita (32). Only one study looking at E-Vita Open device reported a relatively low dSINE occurrence of 1% of a patient population of 286. However other complications such as endoleak occurred in 5% of patients which is more than any study investigating Thoraflex™ Hybrid (33).

Berger et al. (24) also directly compared Thoraflex™ and E-Vita Open and further proved Thoraflex Hybrid's more favorable results as 14.5% of patients in the Thoraflex Hybrid™ developed dSINE post-FET relative to 18.2% with E-Vita Open (*P* = 0.19). To extend this claim, a dSINE rate of 7.1% was reported by Fiorentino et al. (20) in their study which used Thoraflex Hybrid™ in 28 patients with chronic dissections/post-dissection aneurysms.

Custom-made graft and J Graft Open Stent Graft (Frozenix™) studies have reported relatively positive dSINE results, however, these are widely varied between studies. Hirano et al. (34) reported that dSINE occurred in 4% of J Graft Open Stent Graft patients. Again, a replicated study found that dSINE occurred in only 1 patient (0.72%) with the J Graft Open Stent graft which required TEVAR reintervention (35). Interestingly, none of the patients 50 in Iino et al. (36) suffered from dSINE post-FET. However, the authors here used the Frozenix graft in combination with the Gelweave™ graft (Terumo Aortic, Scotland, UK). This shows the extent of the positive affect induced by the Terumo Aortic line of aortic arch prostheses (36). On the other hand, Yamane et al. (37) also used Frozenix™ HP alone in 11 patients out of whom 3 developed dSINE (27.3%). Another study reported 9 cases of dSINE amongst 70 patients receiving Frozenix™ HP (12.9%). All 9 patients underwent successful reintervention with TEVAR (25). Finally, Furutachi et al. (31) reported a dSINE rate of 15.8% amongst their Frozenix™ FET group. Spring back force from the stent graft has been established to be one of the underlying mechanisms for dSINE, causing injury to aortic intimal when the spring force straightens its configuration (27). Furutachi et al. (31) also stated that the Frozenix™ graft is said to exert a strong spring back force on the aortic wall which increases the risk of dSINE developing. On the contrary, the Thoraflex Hybrid™ design has been shown to actually reduce the stress on the aortic wall (38).

Very surprisingly, despite a comprehensive and thorough literature search using multiple databases, no evidence on dSINE directly associated with Cronus™ could be identified. Tan et al. (39) is the only FET study identified which used Cronus™ and reported on untreated entry tears (63.6%), however, these were distal to the stent graft itself therefore not directly induced by it. It is worth noting that Cronus™ is geographically confined mainly to China, hence why there is little to no data on it making it difficult to draw a definitive conclusion on its effectiveness regarding dSINE (38).

If dSINE is diagnosed, the initial management involves optimizing blood pressure. If the false lumen continues to grow larger in diameter, pseudoaneurysm formation, malperfusion or development of symptoms occur then secondary endovascular reintervention with TEVAR is necessary. The artery of Adamkiewicz is identified in the preoperative CT and preserved to the best ability to prevent the risk of paraplegia. Secondary reintervention with TEVAR for dSINE has been reported to achieve excellent results (25, 40).

Several preventive procedures can be undertaken to prevent the development of dSINE, firstly endografts with tapered configuration can potentially be used to prevent excessive oversizing. This tapering effect in diameter and area comprises of using a small stent graft distally and a larger stent graft proximally (38). In TEVAR, Hsu et al. (41) demonstrated dSINE occurrence decreased from 34.7 to 8.3% using this endografting technique. This was also supported by Janosi et al. (42), who also demonstrated the effectiveness of this TEVAR technique in reducing the incidence of dSINE. Janosi et al. (42) found that patients who developed dSINE showed a significantly higher taper ratio of the true lumen of the aorta (40.9 ± 14.13% vs. 25.36 ± 20.2%, *p* < 0.05). The same principle applies in FET as proven by Ma et al. (38), who described tapered stent grafts as the “ideal” ones. In fact, this same fascinating review indicated that Thoraflex™ Hybrid is the only FET HP available commercially with a different proximal and distal diameter of its stent graft portion. Unlike other FET devices, Thoraflex™ Hybrid is unique in that is has a wide and versatile portfolio with varying combinations of stent-graft diameters as well as stent graft lengths. In addition, as aforementioned its interrupted pattern projects minimal radial forces on the diseased aortic wall relative to the other aortic arch hybrid prostheses, confirming Thoraflex Hybrid™ to be the optimal FET device choice for TAR (13, 38). It is essential to correctly choose the FET graft size after careful TL measurement, to avoid aortic remodeling mismatch in the distal transition zone between the stent-covered aorta and non-stent-covered aorta, thereby minimizing the risk of dSINE developing and maximizing the rate of aortic remodeling.

## Is Aortic Remodeling Correlated With Dsine?

The culmination of the process of aortic remodeling is marked by the complete thrombosis and elimination of the FL with the normalization of the TL diameter. This achieved by the action of the stent graft portion of the hybrid prosthesis (HP) sealing off any entry tears and cutting the perfusion to the FL (29, 30). Developing dSINE means the FL remains perfused and patent, thereby negatively influencing aortic remodeling, this concept has been discussed in several studies (29, 30). Nomura et al. (25) stated that all patients who developed dSINE also developed enlargement of the thoracic aortic diameter, including the FL, whereas thoracic aortic remodeling did not occur. This is significantly incomparable with the 57.3% thoracic aortic remodeling rate in the non-dSINE group (*p* = 0.0013). Furthermore, a Russian single-center study (32) reported that 11% of their FET patient population who experienced dSINE also showed negative aortic remodeling which necessitated reintervention with thoracic endovascular aortic repair (TEVAR). In addition, Wada et al. (27) stated that the non-stent-covered aorta is unlikely to undergo sufficient remodeling due to the expansion of the FL and the pulsatile wall stress in the FL, both caused by dSINE. Another study which showed an identical trend is Kreibich et al. (9), which found that patients who developed dSINE showed to have smaller true lumen diameters at the level of the stent graft (L1; *p* = 0.251) and the level of thoracoabdominal transition (L2; *p* = 0.44). To further prove the relationship between dSINE and aortic remodeling, both Huang et al. and Chen et al. (43, 44) conducted studies on TEVAR in type B aortic dissection (TBAD). Results demonstrated that patients who suffered from dSINE post-TEVAR also showed significantly worse aortic remodeling.

## Fet Insertion Length, Graft Size, Aortic Remodeling, and Dsine: Reality Or Myth?

Aortic remodeling and dSINE are connected together, but might also be associated with a FET graft length and size, as some studies study revealed that these two variables strongly influence both of the rate of aortic remodeling and the incidence of dSINE, both concomitantly and independently.

When it comes to aortic remodeling and dSINE associated with FET, in addition to the difference between anatomical sites and the presentation of the dissection, another significant factor to consider is the distal landing site for the prosthetic anastomosis. The landing zone in the FET technique has historically been zone 3, at the proximal descending aorta, after the left subclavian artery. However, novel evidence has suggested that zone 2, between the left common carotid and subclavian artery, yields more benefit (23). The Society of Vascular Surgery/Society of Thoracic Surgeons (SVS/STS) Aortic Dissection Classification System of dissection subtype according to zone location of primary entry tear is well-illustrated in Lombradi et al. (45). Firstly, a 2019 study of 282 patients reported that proximilisation of the anastomosis led to a reduced visceral ischaemia time (*p* = 0.001) and other complications such as recurrent laryngeal nerve injury (46). Another 2018 study supported this by evidencing significantly lower rates of post-FET renal (*p* = 0.004) and pulmonary failure (*p* < 0.001). Additionally, these benefits were observed long-term, where the 5-year survival rates were also superior with zone 2 implantation (*p* = 0.022) (33). Therefore, we thought it would be of excellent academic merit to evaluate whether aortic remodeling rates would also be influenced by the distal landing zones of the anastomosis, to improve future practice and establish its connection to both aortic remodeling rate and dSINE incidence.

Despite the overarching principal that proximilisation of the distal anastomosis offers superior results in general, a study by Panfilov et al. (23) describes zone 2 anastomosis as a “double-edged sword.” This 2021 study concluded that proximilisation decreases the extent of the FET procedure, in addition to the ischaemic time, and overall post-operative morbidity and mortality (23). However, complete FL thrombosis was significantly higher with zone 3 anastomosis. At 24 months post-FET, FL thrombosis in zone 2 was 60 and 77% in zone 3 (*p* = 0.046). Additionally, this increased remodeling was thought to explain the lower reintervention rates in zone 3 (8.3 vs. 25.9%). Panfilov et al. (23) hypothesized that failure of FL thrombosis may be due to the reduced length of coverage in the aorta, causing the higher rates of reintervention. This is congruent to Berger et al. (24) findings that aortic remodeling could be improved with longer coverage of the descending aorta and hence further stabilization.

This notion to use longer FET stent grafts was also fully supported by Yamane et al. (37), who discovered a strong association between the FET insertion length and dSINE incidence as a short insertion length was much more common in the dSINE group, thereby further confirming our earlier statement on this relationship. However, for future practice using the Thoraflex™ Hybrid, Leone et al. (46) made a different recommendation of using the shorter graft length of 100 mm for better aortic remodeling outcomes. The authors suggested that the 100 mm endograft can sufficiently stabilize the dissection flap and that a large intimal tear is often located proximally near the left subclavian artery. Moreover, a recent study has shown that elongated grafts were associated with a 13% increase in freedom from negative remodeling (47). Nevertheless, the majority of evidence favors greater FET insertion lengths for improved remodeling. Regarding graft size, a 2015 review noted that oversized stents in acute dissection could attribute to new intimal tears and subsequently impair aortic wall healing and remodeling (12, 21). As shown in Table 4, varying lengths and diameters of the Thoraflex™ Hybrid were used in different studies.

Kreibich et al. (9) used the 100 mm Thoraflex Hybrid™ graft used in 123 patients, 14 (11.4%) developed dSINE, and out of 3 patients treated with the 150 mm graft, 2 (66.7%) developed dSINE. The authors also found that larger stent graft diameters and oversizing were more common in dSINE patients. This is another study that supported using longer FET stent grafts to increase the insertion length and extend the stent graft coverage of the aorta more distally, to reduce the risk of dSINE in this case, in addition to Berger et al. (24), Yamane et al. (37), and Panfilov et al. (23) mentioned above. On the other hand, Nishi et al. presented a counter argument that some surgeons recommend using a short FET stent to decrease the direct coverage to the segmental arteries and ensure the blood supply to the spinal cord. However, this was countered by Tan et al. (39), who stated that this alone is insufficient to avoid paraplegia and that the stent should be extended distally to treat any distal entry tears. Thoraflex™ Hybrid is licencesed to be implanted across the entire arch (zone 0–4), thus the distal anastomosis zone will determine the length of graft needed (48).

The compelling evidence in this review strongly establishes the connection between dSINE, aortic remodeling, and the insertion length of the FET stent graft. Kreibich et al. (9), Yamane et al. (24), Berger et al. (37), Tan et al. (39), and Panfilov et al. (23) alls agreed that using longer FET stent grafts is very likely to reduce the incidence of dSINE or boost aortic remodeling. With the proven negative relationship between the latter two, the nature of the relationship between these three variables is a three-way one.

Excessive oversising of the FET graft distally has also been strongly associated with dSINE occurrence. Several studies have established that oversizing is a risk factor for dSINE development, however, a few other studies have argued against this. Di Marco et al. (1), Wada et al. (27), Berger et al. (24), Tsagakis et al. (28) and Canaud et al. (49) all recommended against unnecessary graft oversizing as they argued that excessive oversizing is a significant risk factor for development of dSINE. Janosi et al. (42), Jang et al. (50) and Huang et al. (51) studied the relationship between distal oversizing and dSINE and presented solid evidence showing a significant difference in oversizing ratios between patients who did and did not develop dSINE, further extending the validity of the above argument. On the other hand, although Kreibich et al. (9) also recommended avoidance of oversizing, there was a numeric but not statistically significant trend toward oversizing in their dSINE patient group (*P* = 0.613). In addition, in contrast to the majority of reports both Nomura et al. (25) and Yamane et al. (37) did not find a significant different in oversizing between patients with and without dSINE post-FET. Finally, Tochii et al. (3) presented both sides of the argument by stating that although graft oversizing may induce intimal damage, undersizing the FET stent graft may also increase the risk of type 1b endoleak which can negatively influence aortic remodeling through impeding FL thrombosis. Damberg et al. (52) also supported the use of 10–20% oversized grafts to reduce the risk of type Ib endoleak by allowing a tighter distal stent-graft anastomosis. It is worth noting the studies highlighted used different definitions and calculations for oversizing. Yet, all evidence considered, it is clearcut that excessively oversizing the stent-graft, particularly distally, significantly increases the risk of forming dSINE. Given the already established relationship between dSINE and aortic remodeling, it is safe to say that FET graft size is in turn associated with aortic remodeling.

## Conclusion

In conclusion, aortic remodeling has been a significant indicator for patient prognosis following the FET procedure. The Thoraflex™ Hybrid device has evidenced significantly increased TL diameter, decreased FL diameter, and favorable FL thrombosis in both CT angiography and volumetric measurement studies. Moreover, these effects were superior to the other HP available commercially and were also maintained long-term. Not only does the Thoraflex™ Hybrid show excellent aortic remodeling, but it has also shown reduced dSINE rates in comparison with other HPs, encouraging further studies into the effectiveness of this device and its increased implementation in future practice given its favorable outcomes and high versatility. With dSINE negatively influencing aortic remodeling distally and with FET insertion length interconnecting with both outcomes, the choice of both FET graft size and length must be made with great care to achieve optimal results.

## Author Contributions

MJ, FK, PS, and DA were involved in literature review design, literature search, and manuscript writing. YR was involved in literature search. ST, MM, SH, IM, and MB involved in manuscript revision. All authors contributed to the article and approved the submitted version.

## Conflict of Interest

The authors declare that the research was conducted in the absence of any commercial or financial relationships that could be construed as a potential conflict of interest.

## Publisher's Note

All claims expressed in this article are solely those of the authors and do not necessarily represent those of their affiliated organizations, or those of the publisher, the editors and the reviewers. Any product that may be evaluated in this article, or claim that may be made by its manufacturer, is not guaranteed or endorsed by the publisher.
